# Reliability of Cycle Applications for Pregnancy Planning and Contraception: A Systematic Review

**DOI:** 10.1016/j.mcpdig.2025.100239

**Published:** 2025-06-09

**Authors:** Isabell Rabe, Jan P. Ehlers

**Affiliations:** a Didactics and Educational Research in Healthcare, Medicine Department, Faculty of Health, Witten/Herdecke University, Germany; b Public Health, Professional School, Leuphana University Lueneburg, Germany

## Abstract

**Objective:**

To show the effectiveness of cycle applications in both areas of application—contraception and intended pregnancy.

**Methods:**

A systematic review based on the PubMed and Google Scholar databases, with the addition of a hand search, was conducted from May 11, 2023, through April 11, 2024, to objectively answer this question. Of 1539 sources with matching search terms, 19 sources remained after checking for inclusion criteria according to the Preferred Reporting Items for Systematic Reviews and Meta-Analyses scheme. These were analyzed according to an evaluation scale regarding their quality in various areas. The average quality ratings and pregnancy probabilities of the studies were compared.

**Results:**

Comparability within and between the subquestions was hardly possible owing to different presentation of results, bias risks, and mostly uncontrolled study designs. Applications for those wishing to become pregnant provided better quality ratings in some cases. There were indications that cycle applications shorten the time to achieving a desired pregnancy in cases of reduced fertility. In addition, some seem to have a similar contraceptive safety as the contraceptive pill but require significantly more compliance.

**Conclusion:**

Independent, controlled studies with a diverse clientele of test subjects are necessary for a scientific classification. In addition, social, structural, and political adjustments are needed to enable individuals to make informed decisions about the use of cycle and fertility applications.

The compatibility of contraceptives is becoming increasingly important, especially for young people.^1^ In the German health care system, these informed and empowered individuals with a desire for alternatives to hormonal contraception often encounter practitioners who are fundamentally sceptical about information obtained from the internet and who attach great importance to contraceptive safety, which is why concerns about possible side effects tend to be met with a placatory response.^2^^,^^3^ Consequently, patients frequently look for alternative, self-determined contraceptive methods, just as couples with an unfulfilled desire to have children look for support. Fertility applications for determining the fertile period are attracting customers for both areas of application. However, to the best of our knowledge, there is no legal classification in Germany that is safe for users.^4^ The Association of the Medical Societies in Germany has established a guideline addressing nonhormonal contraception, which was published in 2024.^5^ The varying effectiveness of various methods of natural family planning were mentioned, as well as the lack of sufficient evidence supporting the effectiveness of contraception applications, regardless of their symptothermal basis. It is not uncommon for users to be misled by the false sense of security that is created by relying on the known certifications of the applications in question.^6^ However, according to Frank-Herrmann et al,^7^ an approval of some contraceptive applications by Conformité Européenne (European Conformity) classification, Technische Überwachungsverein (Technical Inspection Association) seal, or US Food and Drug Administration is not a guarantee of their safety of use. This is because these organizations do not test their effectiveness in their own studies; instead, they rely on the information and documents provided by the manufacturers. Furthermore, numerous applications are not designed for contraceptive purposes but are used for this function based on the stated fertile period.^7^

### Natural Family Planning

The term natural contraception is not always unambiguous for users and professionals alike.^8^ According to the latest guideline,^5^ in order to achieve a high level of contraceptive effectiveness, only symptothermal methods with a high level of effectiveness should be recommended. One evidence-based method is marketed under the brand name Sensiplan.^9^ The beginning and end of the fertile phase can be determined by observing the hormonally triggered, ovulation-related rise in basal body temperature and the consistency and color of the cervical mucus (or the consistency and position of the cervix), through a double check.^10^ However, it should be noted that other measurement times or types, alcohol, illnesses, physical activity, traveling, heat, or hypothermia can influence this.^11^ Other factors that may influence the menstrual cycle include physiological cycle fluctuations^12^ and the impact of age, body mass index (BMI), and ethnicity on the cycle.^13^ Most users express a preference for evidence-based contraceptive applications.^14^^,^^15^ However, although many applications claim to predict fertile days and thus be usable as a safe contraceptive method,^16^ only a few are based on evidence.^14^^,^^15^

### Possible Ineffectiveness

Cycle applications provide users with a high degree of empowerment. However, users frequently lack the requisite health literacy to reliably categorize the information they receive, for example, the reliability of the specified fertile period, which can result in unintended pregnancies.^17^ Furthermore, numerous cycle applications lack substantial medical sources and are not developed by medical professionals,^18^ and the prediction is often inaccurate.^19^^,^^20^

### Objective and Research Question

To show the effectiveness of cycle applications in both areas of application, the research question was as follows: “How reliable is the prediction of the fertile window by menstrual cycle applications for users who are planning a pregnancy by using such an application, and how reliable is the prediction of the fertile window by menstrual cycle applications for users who are using such an application for contraception?”

## Methods

The method of the systematic review was selected to ensure the greatest possible objectivity in answering the research question.^21^ This systematic review was conducted from May 11, 2023, through April 11, 2024. This was achieved by minimizing potential bias through a meticulous, preplanned procedure in accordance with the *Cochrane Handbook for Systematic Reviews of Interventions*. To guarantee a systematic and reproducible search for pertinent sources, the literature search was meticulously aligned with the criteria set forth in the PRISMA (Preferred Reporting Items for Systematic Reviews and Meta-Analyses) statement.^22^

As inclusion criteria, we searched for studies that dealt with smartphone cycle applications that support users based on the cycle information entered and the fertile time calculated from it, either for contraception or in the event of pregnancy. Because both achieving pregnancy and contraception based on cycle monitoring are closely linked to determining the correct time of ovulation, the broader concept of the time of ovulation was also included in the inclusion criteria.

The exclusion criteria were related areas with a different focus as follows:

• Studies on animals, microorganisms, plants, or fungi
• Applications that record the measurement of luteinizing hormone in urine, for example, as the main measurement parameter
• Stand-alone cycle computers without applications
• Applications for preconception care
• Applications for pure knowledge transfer without cycle evaluation
• Applications that provide information on pregnancy (eg, visualization of growth)
• Cycle monitoring only in relation with certain diseases (eg, sexually transmitted diseases, premenstrual syndrome and premenstrual dysphoric disorder, polycystic ovary syndrome, diabetes, and tumors)
• Cycle monitoring only in relation with certain medications
• Applications specifically for people undergoing fertility treatment
• Applications that are explicitly aimed at people in menopause or puberty
• Studies that summarize applications and do not break them down individually by name
• (Partial) publications that refer to a study that has already been included
• Studies that examined applications purely technically on hypothetical cycles

Based on these defined inclusion and exclusion criteria, the first database search was conducted in PubMed (May 12, 2023) and Google Scholar (June 9, 2023), supplemented by a hand search and repeated search until the end of the study. The search terms included the keywords *cycle application* and *contraception*, as well as *cycle application* and *desire to have children* (and the German translations thereof). The languages selected for the search were German and English. As the term *cycle tracking* applications only emerged in 2015,^23^ no temporal constraints were imposed on the search. The databases searched and the respective result figures are shown in Supplemental Appendix 2 for reference (available online at https://www.mcpdigitalhealth.org/). Supplemental Appendix 3 (available online at https://www.mcpdigitalhealth.org/) presents the search generation process for PubMed search.

**Figure 1:**
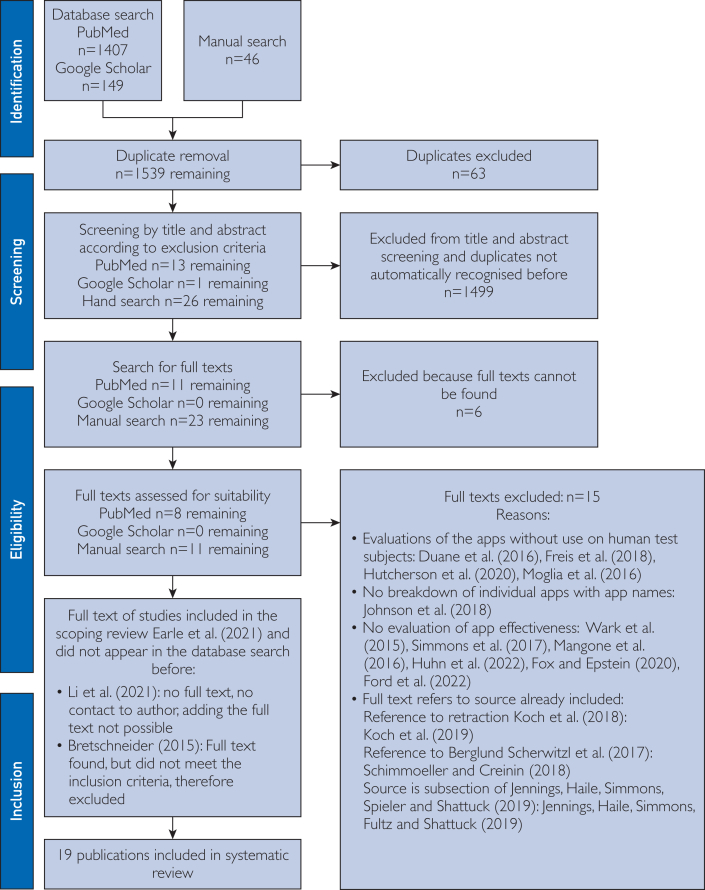
Flow chart based on PRISMA (Preferred Reporting Items for Systematic Reviews and Meta-Analyses) scheme according to Ziegler et al.^22^

**Figure 2:**
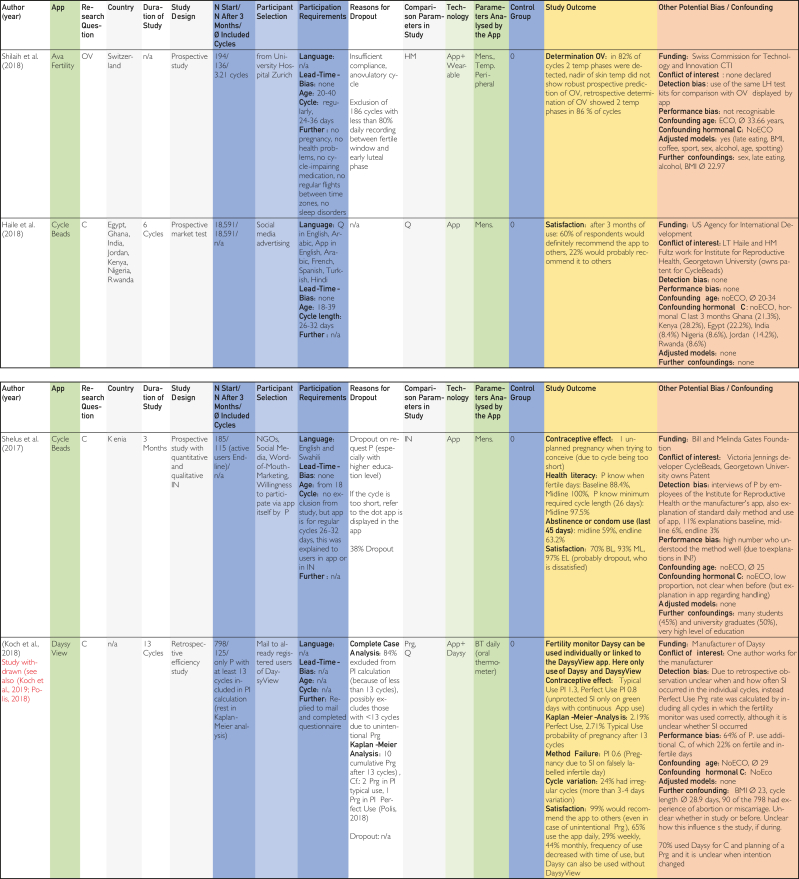

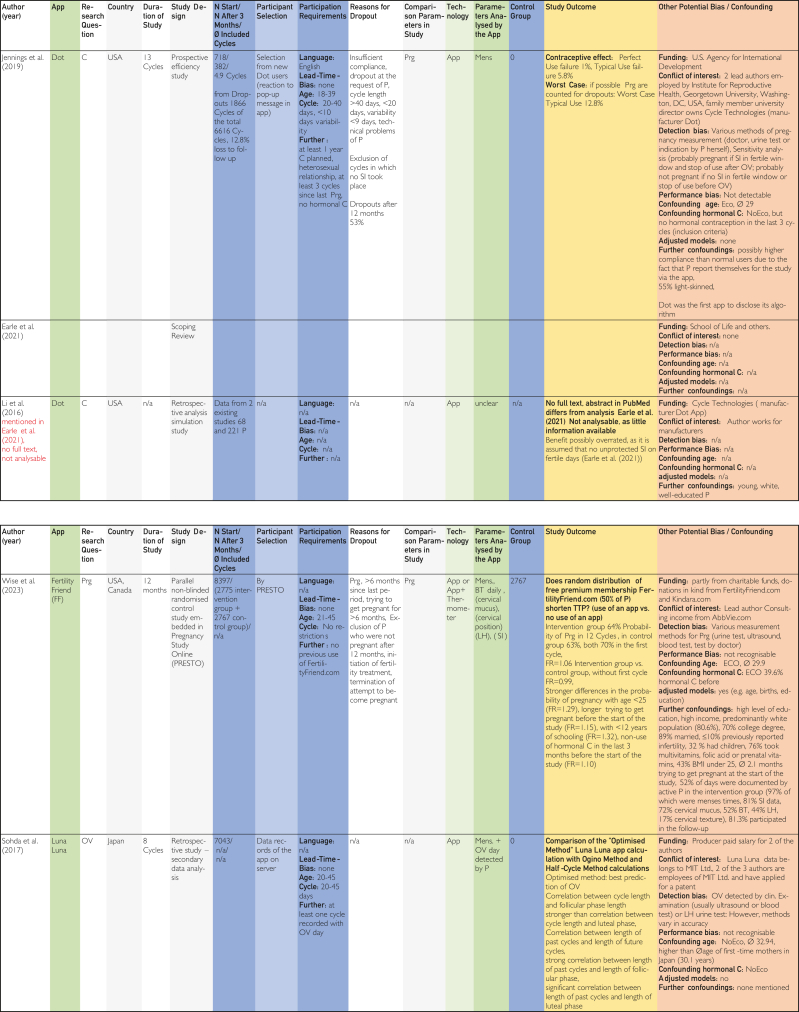

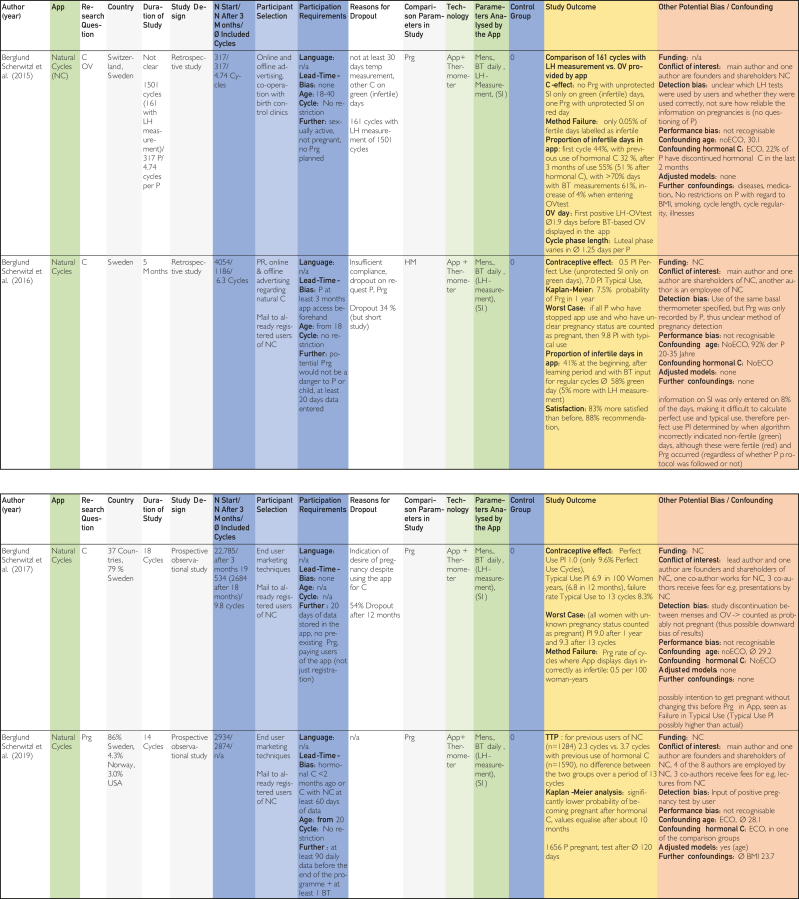

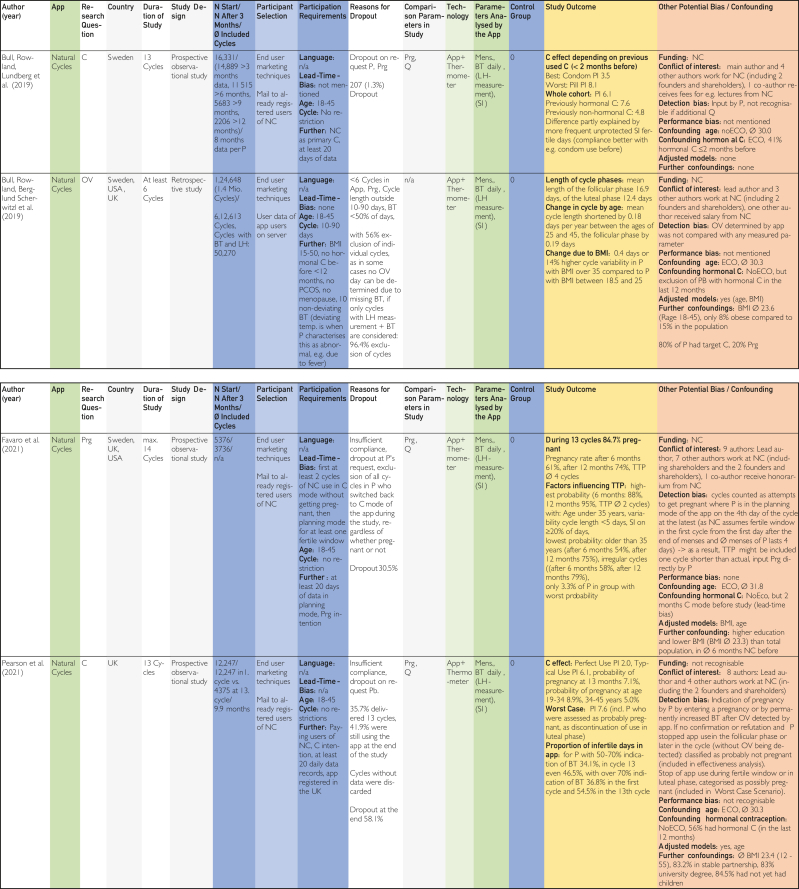

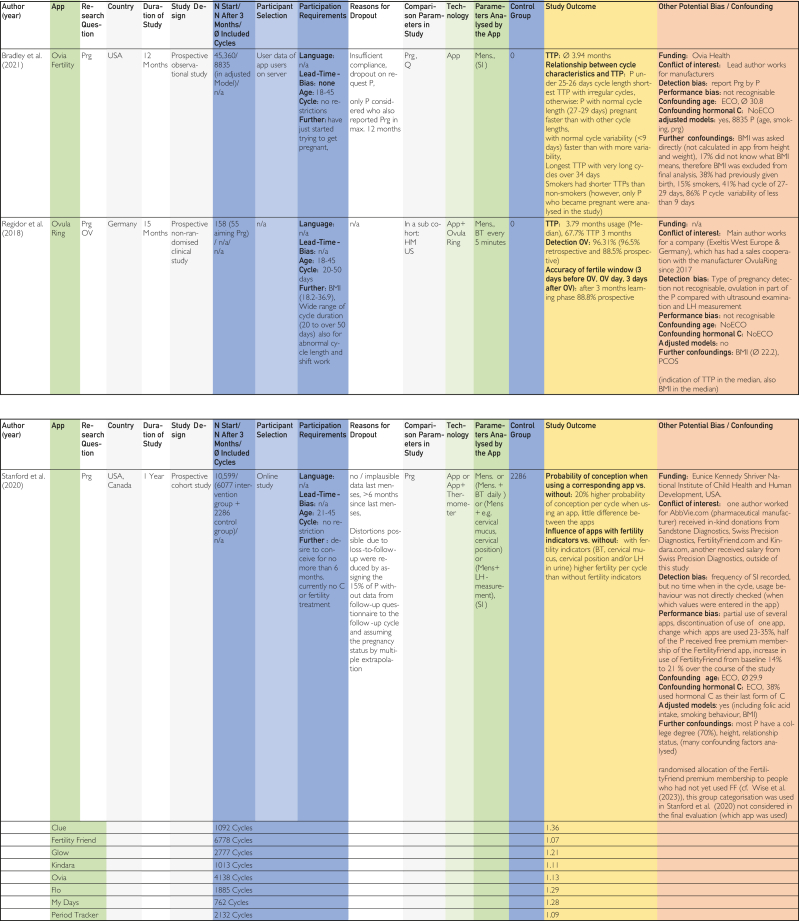
Data Extraction Table (Sorted by App Name).

This was followed by duplicate removal and screening by title and abstract according to the inclusion and exclusion criteria in accordance with the PRISMA statement as shown in the PRISMA Checklist in Supplemental Appendix 1. Full texts were searched for the remaining studies, and these were also screened individually by both authors (Figure 1).^22^ The included publications identified in this way were analyzed in a data extraction table Figure 2 24, 25, 26, 27, 28, 29, 30, 31, 32, 33, 34, 35, 36, 37, 38, 39, 40, 41, 42, 43, 44, 45 using previously defined quality characteristics and then evaluated in tables on the topics contraception, pregnancy, and time of ovulation according to a defined quality assessment key. The resulting average quality scores and pregnancy probabilities were then compared.

### Included Publications

**Figure 3:**
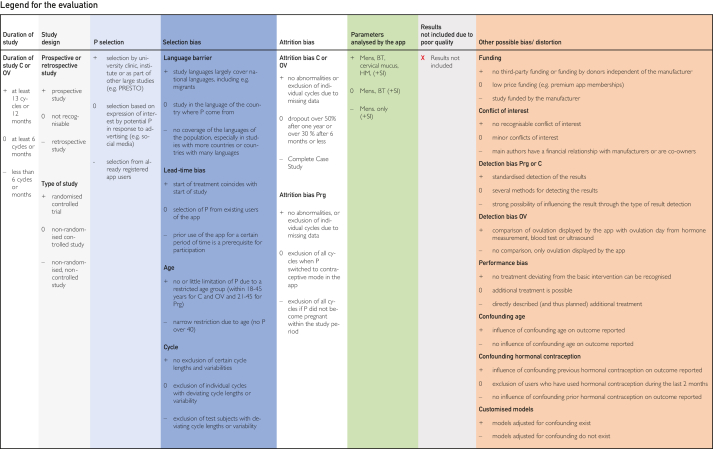
Legend for Tabular Analysis of the Topics Contraception, Pregnancy and Ovulation Time.

**Figure 4:**
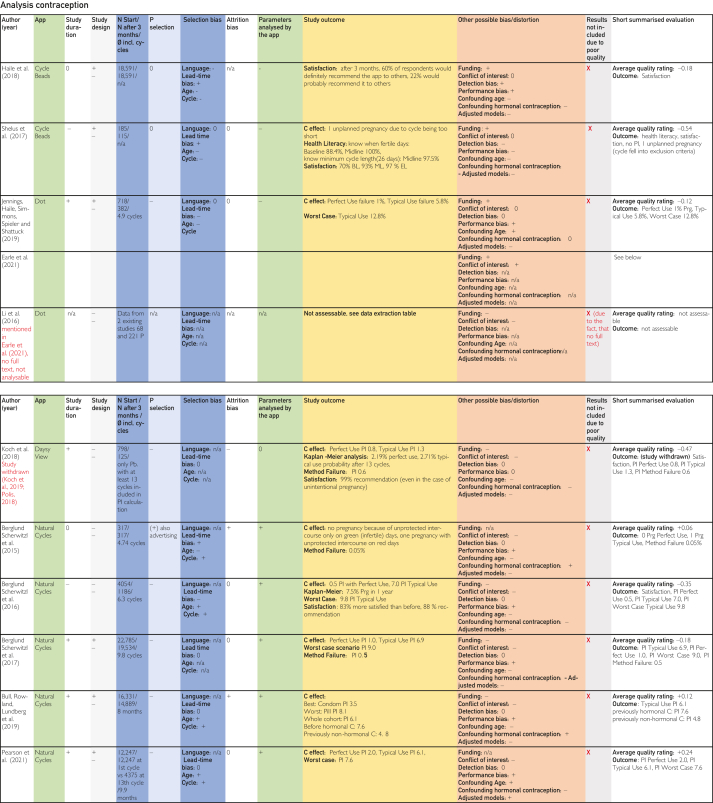

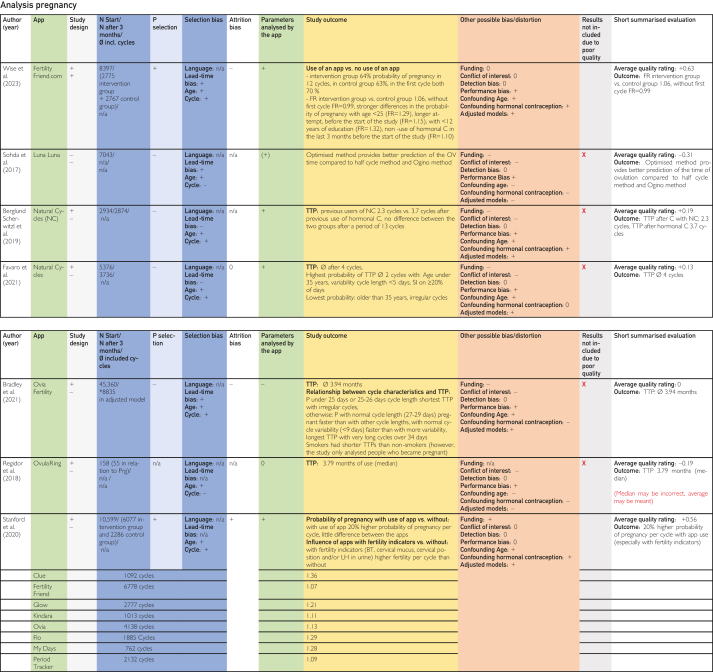

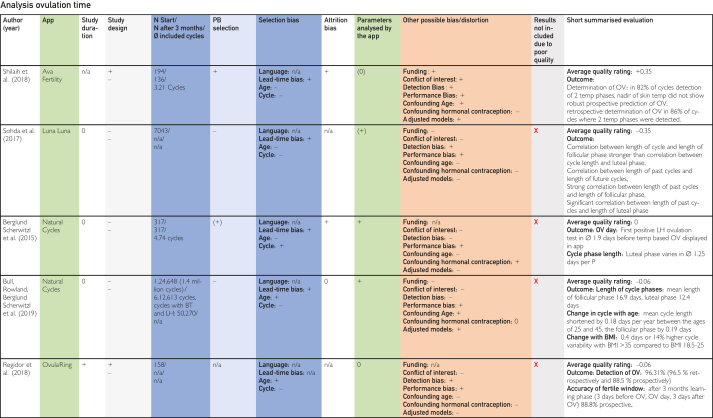
Tabular Evaluation of the Topics Contraception, Pregnancy and Ovulation Time

The categories delineated in the columns are based on the Cochrane PICOS (population, intervention, comparison, outcomes, study) scheme. The population section includes the columns “No. of cycles at start/No. of cycles after 3 mo/∅ included cycles,” “Participant selection,” and “Participant requirements.” The columns “Application,” “Technology,” and “Parameters analyzed by application,” on the contrary, pertain to intervention. The column designated “Control group” is categorized at comparison. The outcome is delineated in the “Study outcome” column. The study type is described in the column “Study design.” Abbreviations used in the tables in Figure 2, Figure 3, Figure 4 24, 25, 26, 27, 28, 29, 30, 31, 32, 33, 34, 35, 36, 37, 38, 39, 40, 41, 42, 43, 44, 45 are explained in the list of abbreviations in Supplemental Appendix 4 (available online at https://www.mcpdigitalhealth.org/). The data extraction table can be found in Figure 2. The assessments in Figure 4 were performed according to the legend in Figure 3

### General Issues Encountered During the Course of the Evaluation

Owing to the lack of randomized controlled trials and the heterogeneity of the included studies in terms of methods, calculation methods, follow-up conditions, and lost-to-follow-up rates, as well as the inadequate sample sizes and considerable risk of bias, the quality of most of the included studies was found to be inadequate. That is why, most studies (with the exception of 3) found deficiencies in internal and/or external validity, resulting in the inability to attain objective comparison. As a result, the results of 16 of the 19 included and evaluated studies could not be considered valid because of various potential biases or limitations in generalizability and were excluded (Figure 4 “Results not included due to poor quality”).

It is also important to note that of the 19 included studies, 8 studies addressed the application Natural Cycles, 5 studies assessed the contraceptive effectiveness, and 3 assessed the topic of pregnancy. Although not all of them are written by the same authors, these studies appear at least similar and low in quality owing to strong risks of bias because of manufacturer funding, several conflicts of interests, and deviating methods of calculations.

Two studies^33^^,^^45^ were identified with adequate quality. These studies were controlled and not funded by producers, addressing the topic of pregnancy. However, given that both were part of the Pregnancy Study Online, a cohort study from North America, it is not reasonable to compare them. A third study^24^ also reported good quality, but a direct comparison was not possible owing to a lack of data regarding pregnancy rates. Instead, the discussion centred on the potential of a bracelet that measures body temperature to predict ovulation.

Nevertheless, it is important to articulate these limitations because they are not always immediately obvious on initial inspection of the studies’ documentation available on the internet. Our quality rating is displayed in the following sections and in Figures 3 and 4. At this point, it is important to acknowledge that a comparison of the included studies was not possible owing to these huge differences in the quality of the studies. Because of the heterogeneity of the included studies, it was not possible to address and assess all potential shortcomings and biases we found in this study. It is also important to emphasize that the effectiveness of the studies outlined in the following sections, being calculated differently, are subjects to various limitations, including limitations in generalizability and potential biases. Therefore, a comparison was not possible, and the effectiveness described in these studies might not be transferable to the heterogeneity and diversity of potential users. In the following sections, we outline these values and the limitations of these studies.

### Summary of Evaluations on Contraception

The average quality ratings were formed based on the evaluations. The lowest quality rating was evaluated at the study done by Shelus et al.^26^ Primary factors contributing to this were potential bias resulting from the use of CycleBeads being explained to study participants during interviews and only 37% of the 185 participants used CycleBeads for contraception, as well as the short study duration of 3 months.

The second lowest quality rating was found in the study by Koch et al,^27^ which was retracted.^29^ A further 4 studies reported mixed ratings with more negative ones. Of the 8 studies included in this article that address the application Natural Cycles, 5 studies assessed the contraceptive effectiveness of this application. A mere 3 studies yielded positive averages, led by Pearson et al,^42^ but as already mentioned, the quality was still poor (see General issues encountered during the course of the evaluation section). A comparison of the values described as the contraceptive effect was only possible for 5 sources with Pearl Index (PI) information. The 4 sources referring to Natural Cycles^36^^,^^37^^,^^39^^,^^42^ reported similar typical-use PI between 7.0 and 6.1, with different quality scores ranging from 0.35 to 0.24. The study by Koch et al^27^ with the typical-use PI of 1.3 (which differs greatly from the other 4 studies) was withdrawn by the editor-in-chief of *Reproductive Health* owing to suspected selection bias and retrospective self-reporting by the participants as to whether pregnancy was intended.^29^ This study found even more limitations: the low response rate (only 13%) and the fact that this was a complete case study, which excluded women who participated for less than 13 cycles (potentially resulting in the exclusion of their pregnancies).

Bull et al^39^ compared the typical-use PI of 7.6 after previous hormonal contraception (2 months before the start of the study) with previous nonhormonal contraception (PI=4.8). The entire cohort resulted in a PI of 6.1. For the comparison, the typical-use PI of all test subjects was considered. The lowest PI (of 3.5) was found after previous condom use, the highest after use of the pill (PI=8.1). According to the authors, this could be due to the fact that condoms, similar to natural contraception, require better compliance compared with hormonal contraception. The more frequent unprotected sexual intercourse on potentially fertile days after hormonal contraception speaks in favor of this.

No PI data were available for 4 studies, so a comparison was not possible. Haile et al^25^ reported user satisfaction as an outcome, which was also mentioned by Shelus et al.^26^ However, the topic of user satisfaction was not included in the research question. Furthermore, user satisfaction can be biased by influencing of the participants answers (intentionally or not) or simply by social desirability. Shelus et al^26^ recorded an improvement in health literacy over the course of the study, although this could be due to the way the results were recorded in interviews. As with the other studies with poor quality, the results had to be excluded because only 37% of the 185 participants used CycleBeads for contraception and the duration of this study was only 3 months. Jennings et al^30^ described the perfect-use failure rate (ie, the proportion of probands with unplanned pregnancies) as 1%, whereas in typical use, it was 5.8%. However, only 24% of the cycles were reported as perfect-use cycles. This lacks generalizability and reports the low inherent effectiveness of these methods of contraception because the discrepancy between typical and perfect use is obvious owing to possible errors in use. The low percentage of perfect-use cycles documented in the included studies indicates that there is a significant degree of compliance necessary to ensure the proper utilization of these methods. Berglund Scherwitzl et al^35^ reported no pregnancy (n=0) in participants who practiced unprotected sexual intercourse only on green (infertile) days and 1 pregnancy in unprotected sexual intercourse on days labeled as fertile.

As a worst-case scenario, that is, typical-use including test participants with unclear pregnancy status at dropout assigned as part of the sensitivity analysis, Jennings et al^30^ described the probability of unintended pregnancies at 12.8%, Berglund Scherwitzl et al^37^ at a PI of 9.0, and Pearson et al^42^ at a PI of 7.6. However, the sensitivity analyses were not comparable with each other (similar most of the other reported results of the included studies). As method failure, that is, pregnancies due to fertile days incorrectly labeled as infertile by the application, Koch et al^27^ described a PI of 0.6, Berglund Scherwitzl et al^35^ a pregnancy probability of 0.05% and Berglund Scherwitzl et al^37^ a PI of 0.5. Unfortunately, there was no information in the other studies on method failure.

### Summary of Evaluations on Pregnancy

**Figure 5:**
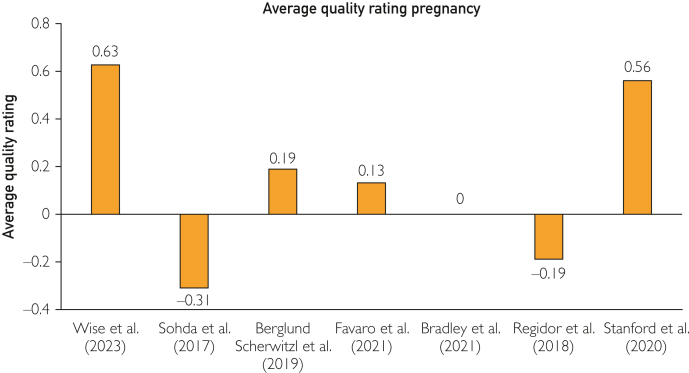
Average quality rating for pregnancy.

Because results of both studies that were not excluded addressed the topic of pregnancy, the overall average quality ratings for contraception were clearly lagging behind (Figure 5). A direct comparison of the time to pregnancy (TTP) was not possible because only the results of the 2 studies by Wise et al^33^ and Stanford et al^45^ were not excluded. Only 4 of 7 excluded studies lined out TTP data. Two of the values were recorded in months, and 2 were recorded in cycles. This makes a comparison of the TTP values impossible because of the natural variations in cycle length. Bradley et al^43^ reported 3.94 months and Regidor et al^44^ 3.79 months, whereas Favaro et al^41^ reported 4.0 cycles and Berglund Scherwitzl et al^38^ reported 3.7 cycles after previous hormonal contraception and 2.3 cycles after previous contraception with Natural Cycles. The Kaplan-Meier analysis in the study by Berglund Scherwitzl et al^38^ found a significantly lower probability of becoming pregnant after hormonal contraception, which approached the values without hormonal contraception after around 10 months. Although the study by Berglund Scherwitzl et al^38^ seem to provide important evidence on the influence of previous hormonal contraception on TTP, the risk of lead-time bias due to previous use of Natural Cycles must be taken into account. There was no comparison group that had not previously used hormonal contraception and was not familiar with the application or natural contraception.

Although Sohda et al^34^ focused on the topic of pregnancy, they compared different calculation methods in relation to the time of ovulation without specifying a concrete TTP or fertility rate (FR). This study had the lowest average quality rating of the included studies on pregnancy applications, followed by Regidor et al.^44^ Favaro et al^41^ reported a lower TTP due to application use in people younger than 35 years of age, low cycle variability, and frequent sexual intercourse. The study by Bradley et al^43^ also seems to support the link between a higher probability of pregnancy and lower cycle variability. However, in the study by Bradley et al,^43^ participants with short cycles of less than 25 to 26 days and with irregular cycles had a higher probability of pregnancy. This could be because shorter cycles have more potential ovulations per year. The fact that Bradley et al^43^ found a lower TTP with application use for shorter cycles with greater variability than that for shorter cycles with less variability could be because in case of short, regular cycles, an erroneous standard 28-day cycle of the application algorithm resulted in the ovulation time being persistently misrepresented. For participants with short but highly variable cycles, the estimated ovulation time point may have been correct, at least in some cycles that happened to last 28 days.^43^ Similarly, Bradley et al^43^ were not conclusive as to why smokers had a shorter TTP. However, this study included in the analysis only participants who became pregnant by the end of the study. So, this complete case study excluded all participants who did not become pregnant.

It was necessary to exclude the results of these studies owing to their poor quality. Consequently, 2 sources remained for this systematic review,^33^^,^^45^ which had the best average quality scores compared with that of the other studies in this systematic review. However, they did not give specific TTP values, but the FR, that is, when a certain subcohort (eg, with application use) achieved pregnancy compared with nonuse. Wise et al^33^ described a very small advantage of the application although it was unclear whether and which fertility indicators provided an advantage. Not only people with a reduced chance of getting pregnant (older than 25 years of age or already trying to become pregnant for a longer period) but also people without hormonal contraception in the past 3 months and with less than 12 years of education found a higher FR through application use.^33^ This influence of school education may indicate an increase in the chances of pregnancy through improved health literacy. Stanford et al^45^ described a 20% higher FR with application use, especially when entering fertility indicators.

### Summary of Evaluations on Ovulation Day

Of the 5 studies included on the additional topic of ovulation day, 1 also described the contraceptive effect^35^ and 2 the effect on participants with the desire to become pregnant.^34^^,^^44^ Shilaih et al^24^ reported the highest average quality score and Sohda et al^34^ the lowest. The outcomes relating to this topic are not comparable with the topics of pregnancy and contraception but provide interesting background information on the menstrual cycle, which was included in the discussion.

## Discussion

### Summarized Evaluation Regarding the Research Question

The findings of this study indicate that a high level of e-Health literacy and compliance is essential for the safe use of fertility applications. Otherwise, users can be misled into a false sense of security regarding the application’s effectiveness. Despite the exclusion of most results, the average of the average quality ratings of the 10 included studies on contraception was 0.16 (SD, 0.25) and for the 7 studies on pregnancy intention was 0.14 (SD, 0.33). In terms of quality rating (despite the exclusion of results), the studies on pregnancy intention predominantly reported better values, which was mainly due to 2 nonproducer-funded, controlled studies.^33^^,^^45^ Because the values can only range between +1 and −1 and there are high SDs, the picture is mixed. In addition, no comparability was possible with regard to the outcome, meaning that the question of whether fertility applications are more suitable for people who want to use contraception or who want to get pregnant cannot be answered conclusively in this study.

### Effectiveness in Persons With a Current Desire to Have Children

Although reproduction is a natural process, reduced fertility can be caused by the increasing age of first-time mothers in industrialized countries and stress.^47^ The average TTP given by the 4 studies on the topic of the desire to get pregnant was just under 4 months and could not be calculated more precisely because 2 studies stated it in months and 2 in cycles, and these results are not evident owing to poor quality of the studies. In addition, the 2 high-quality studies^33^^,^^45^ described inconsistently whether the applications for pregnancy planning generally led to a faster pregnancy. The constant presence of the topic through the application can lead to anxiety and stress, especially among application users who are currently trying to conceive,^41^ with negative effects on the probability of pregnancy.^46^

Although controlled studies are qualitatively preferable,^21^ a comparison with other studies is impossible without information on TTP. According to Hong et al,^48^ the TTP described in the literature without supporting methods (such as determining the fertile period) varies and is influenced by biological, sociological and demographic factors as well as the study design (eg, prospective or retrospective). Eisenberg et al^49^ described the average TTP in the United States in 2020 as 5.4 months, compared with 3 months in 2002. However, only people older than 30 years or those who had previously given birth to at least 1 child reported an increase in TTP.^49^ However, owing to the increasing age of first-time mothers in industrialized nations,^47^ the proportion of individuals aged 30 years and older who express a desire to become pregnant is also on the rise.

### Effectiveness in Individuals With a Contraceptive Intention

Most contraceptive methods have a lower probability of unwanted pregnancies if used correctly and without interruption.^50^ For example, the Standard Days Method on which the Dot and CycleBeads applications are based, in which unprotected sex is avoided from the 8th to 19th day of the cycle, reported a perfect-use PI of 4.75 for regular cycles.^51^ This number is likely to be considered a high probability of pregnancy by many users. However, the typical-use PI was even much higher at a PI of 11.96.^51^ According to Trussel and Grummer-Strawn,^52^ only the influence of imperfect use on the contraceptive effect shows the forgiveness of usage errors by the method without unintended pregnancies occurring. The typical-use values of methods with little room for maneuver for the user (eg, copper or hormone intrauterine devices and hormone implants) are significantly closer to the perfect-use values because there is a high inherent effectiveness owing to the reduction of error factors. In contrast, natural contraceptive methods sometimes show large differences in this aspect.^52^

Even if we disregard the questionable quality of these studies, the difference between the typical-use and perfect-use PI values of these publications ranged between 4.1 and 6.5 and between 9.3 and 5.6 when considering the worst-case typical-use PI. This indicates a lower inherent effectiveness of the methods. This could be because there are more possible sources of error due to the users themselves, for example, when measuring basal body temperature or determining the quality of cervical mucus. Lifestyle also influences cycle variability,^13^ which can affect the accuracy of determining the fertile period.

Moreover, if the quality of the included studies had been high enough to produce results of stronger evidence, the influence of imperfect use on the number of unintended pregnancies would be roughly comparable with the values described by Trussel^50^ for hormonal contraceptive methods that must be used regularly by the users themselves, for example, contraceptive pill or NuvaRing (perfect-use PI of 0.3 vs typical-use PI of 8.0). In contrast, the inherent effectiveness is higher for the usage of hormonal or copper intrauterine devices because of the reduced likelihood of application errors, that is, less user compliance is required to ensure safe use.^50^ The high rate of dropouts and the low percentages of perfect-use cycles (eg, only 9.6% of the cycles in Berglund Scherwitzl et al^37^) appear to support this.

### Influences on the Generalizability of Study Results

Methodologically, there is often a lack of randomization in the available studies, which reduces the transferability of the study results.^50^ The different study durations also greatly reduce comparability as the probability of pregnancy per cycle decreases: the greatest chance (30%) is present in the first cycle, compared with 1% to 3% per cycle after more than 3 years.^47^

The data on user satisfaction of the contraception applications were not objective, but it is important to state that these data seemed very high at 60% to 99% but were very prone to bias (whether intentional or unintentional). These high values are not consistent with the sometimes very high dropout rates. These facts should be kept in mind for individuals interested in natural contraception and tend to rely in advertisements or studies of poor quality. In addition, the question arises as to whether participants have sufficient digital health literacy (e-Health literacy) and compliance^53^ to be able to adequately understand opportunities and risks and use the methods correctly in the long term.^50^ In the context of attrition bias, dropouts can lead to the effect of the intervention being overestimated or underestimated because dropouts can be related to the method used itself.^54^

The number of participants varied from 55 to over 1,000,000. Nevertheless, the inclusion of a considerable number of participants does not inherently ensure comprehensive diversification of the overall cohort. It must be acknowledged that the results may not be considered representative of the broader variability observed in the population.

Only 1 study excluded potential participants for whom pregnancy would pose a risk to mother or child.^36^ Health consequences of unplanned pregnancies should be assessed anamnestically by medical study staff before the start of the study and communicated to participants in an understandable way, as their e-Health literacy cannot be assumed across the board.^53^^,^^55^ Because the participants in most of the studies (n=11) were selected from already registered application users, the clientele was limited to technologically savvy people with basic knowledge of the application in the sense of self-selection (and a risk of lead-time bias). Only 2 sources used clinics to recruit participants,^24^^,^^35^ whereas 1 study used nongovernmental organizations as multipliers.^26^ In contrast to the potential lead-time bias mentioned earlier, 40% of the participants in the study conducted in Kenya by Shelus et al^26^ had never used contraception before. Whether this was due to structural, religious, or financial reasons is unclear; a lack of education on contraceptive issues in some emerging and developing countries could also be a reason.^56^

Education, social status, and cycle variability also appear to be correlated. A lower social status is often associated with a lower level of education^57^ and more cycle variability^58^ with negative effects on the contraceptive effect. In addition, Shelus et al^26^ described higher dropout rates with a higher level of education. The underrepresentation of ethnic groups^59^ is also evident in this systematic review because most of the studies were conducted in industrialized countries. Only 2 studies were conducted in emerging or developing countries.^25^^,^^26^ This may limit generalizability because the PI and dropout rates of natural contraceptive methods may differ from country to country.^60^

Physiologically, cycle fluctuations of up to 14 days are possible even in menstruating persons who assume a regular cycle.^12^ As many as 9 of the included studies had no participation restrictions regarding cycle length and variability, whereas 7 had restrictions. Although the age of the participants was only slightly restricted in 11 studies (18-45 years) and only 5 prescribed tighter restrictions, both younger users younger than the age of 18 years and older users older than 45 years were not included in the study results. Because irregular cycles are to be expected in these age groups in particular owing to puberty and the onset of menopause, respectively,^13^ age-adjusted proof of efficacy is particularly important for these user groups. With regard to the desire to have children, the probability of pregnancy is significantly reduced at an older age.^47^ With regard to contraceptive intent, unplanned pregnancy is also associated with increased risks for the pregnant individual and the child, particularly in people younger than 20 years of age^61^ and in people aged 35 and older.^62^

People with a higher BMI can also experience greater cycle fluctuations,^40^^,^^63^ which can have an impact on the likelihood of pregnancy. The generalizability of study results may therefore be reduced depending on the BMI. This topic was inadequately mapped in the studies. Moreover, the effect of previous hormonal contraception on effectiveness was not stated in 10 studies, and 3 sources avoided this by excluding users who had used hormonal contraception in the previous months. It is therefore difficult to transfer and compare the results, especially because the duration in which previous hormonal contraception influences the probability of pregnancy is presented differently in the literature and can be up to 42 months.^38^^,^^64^

Methodologically, the measured parameters of the cycle applications are one of the most important indicators of their effectiveness, but only if these parameters are also included in the calculations of the fertile time of the current cycle.^7^ Unfortunately, in 4 studies, the parameters recorded by the applications consisted solely of the start of menstrual bleeding and the (optional provided) time of intercourse. These applications are mostly based on calendar methods such as the Standard Days Method and, according to Frank-Herrmann et al,^7^ are very inaccurate owing to cycle variability. Unfortunately, however, it is unclear to what extent these parameters were used to calculate the fertile period. Only 1 study disclosed the algorithm,^30^ but this used the Standard Days Method.

Three applications required a special measuring device: these included a temperature measuring arm band,^24^ an intravaginally worn temperature measuring ring,^44^ and a thermometer, which can also be used independently as a cycle computer.^27^ The remaining 16 studies did not describe any specific measuring device required but enabled the input of basal temperature measurements (eg, oral, vaginal, or rectal) or luteinizing hormone measurements. The study situation on peripheral temperature measurement is currently still contradictory.^5^^,^^65^

In terms of study funding, 10 studies were funded by the manufacturers, which can be associated with increased interest in rapid approval and marketing and in study results with high effectiveness.^50^ Conflicts of interest were visible in 13 studies because at least 1 of the main authors worked for the application manufacturers. Although 5 studies appeared at first glance to have a lower risk of conflict of interest, 3 of them reported how researchers from different studies had professional relationships with (at first glance) different applications.^25^^,^^26^^,^^30^

### Limitations

Because of different requirements for search strings in PubMed and Google Scholar, the selection of search terms was difficult. The study types were also very inconsistent. Originally, clinical trials (ideally randomized controlled trials) were to be searched for in order to obtain higher quality results. However, it turned out that the sources contained different types of studies, particularly on the topic of contraception, and that there were hardly any randomized controlled trials. The selection of study types was therefore expanded to obtain a sufficient number of results, but this made the sources difficult to compare and, in some cases, brought with it a considerable risk of bias, resulting in only 2 studies of 19 meeting the quality standards.

## Conclusion

A comparison of the studies was not feasible, precluding a definitive conclusion regarding the applications’ capacity to assist users. The inconsistency in application naming further complicates the decision-making process for potential users and practitioners alike. Consequently, the health care system, its stakeholders, and politicians are confronted with the increasing challenge of responding more effectively and equitably to the desires and concerns of individuals seeking hormone-free contraception and support in achieving pregnancy. This is because younger generations are seeking contraception and childbearing support options that are straightforward and align with their personal needs. An authoritarian approach and paternalism, as evidenced by legislation and the actions of gynaecologists, coupled with the unclear legal classification of applications and the methodological shortcomings of studies examining many contraceptive methods, will not impede this development.

## Potential Competing Interests

The authors report no competing interests.
