# Supplementary material for: Reliability of Cycle Applications for Pregnancy Planning and Contraception: A Systematic Review
Source: Mayo Clin Proc Digit Health. 2025 Jun 9;3(4):100239. doi: 10.1016/j.mcpdig.2025.100239 (PMC12492232; doi:10.1016/j.mcpdig.2025.100239)
Supplement: Supplemental Appendix 2 [file mmc2.pdf]

Appendix 2: Searched Databases with Results:

Database	Search Terms	Results
PubMed	(See Appendix 3)	1407
Google Scholar	"period tracker" "clinical trials" app pregnancy	47
	"period tracker" "clinical trials" app contraception	53
	"ovulation calendar" "clinical trials" app pregnancy	7
	"ovulation calendar" "clinical trials" app contraception	6
	"fertility tracker" "clinical trials" app	26
	"contraception app" "clinical trials"	10
Manual Search		46
