# Supplementary material for: Reliability of Cycle Applications for Pregnancy Planning and Contraception: A Systematic Review
Source: Mayo Clin Proc Digit Health. 2025 Jun 9;3(4):100239. doi: 10.1016/j.mcpdig.2025.100239 (PMC12492232; doi:10.1016/j.mcpdig.2025.100239)
Supplement: Supplemental Appendix 3 [file mmc3.pdf]

Appendix 3: Search Terms, Search Terms Combined and Entire Search String of the PubMed Search

Search Terms

Zyklusapp	Kinderwunsch	Verhütung	Cycle app	Desire to have children	Contraception
Zyklusapp	Fruchtbarkeit	Verhütung	cycle app	fertility	contraception
Menstruationsapp	Fertiliät	Empfängnisverhütung	menstruation app	fertile window	birth control
	fertiles Fenster	Schwangerschaftsverhütung	menstrual app	fertile days	preventing pregnancy
Ovulationsapp	fruchtbare Tage	Empfängnisschutz	period app	desire to have a child	contraceptive
NFPApp	Kinderwunsch	Verhütungsmittel	ovulation app	fertilisation planning	avoid pregnancy
Fertilitätsapp	Periodenapp Befruchtungsplanung	Verhütungsmethode	fertility app	fertilization planning	pregnancy prevention
Fruchtbarkeitsapp	Empfängnisplanung	Verhütungsplanung	menstrual period app	conception planning	fertility control
Familienplanungsapp	Schwangerschaftsplanung	Verhütungsabsicht		pregnancy planning	birth prevention
	Familienplanung	Vermeidung Schwangerschaft	cycle application	family planning	productive control
Zyklusapplikation	NFP		menstruation application	planned pregnancy	prevent pregnancy
Menstruationsapplikation		Empfängnis Verhütung	menstrual application	ovulation prediction	contraceptives
Periodenapplikation	Befruchtungs Planung	Schwangerschafts Verhütung	period application	fertility awareness	avoiding pregnancy
NFPApplikation	Schwangerschafts Planung	Verhütungs Mittel	ovulation application	FABM	
Fertilitätsapplikation	Familien Planung	Verhütungs Methode	fertility application	planned parenthood	
Fruchtbarkeitsapplikation	Schwangerschaft	Verhütungs Planung	menstrual period application	reproductive planning	
Familienplanungsapplikation		Verhütungs Absicht		intentional pregnancy	
		Schwangerschaftsvermeidung	cycle tracker	pregnancy	
Zyklus App			menstruation tracker	ovulation detection	
Menstruations App			menstrual tracker	fertility monitoring	
Perioden App			period tracker	rhythm method	
Ovulations App			ovulation tracker		
NFP App			fertility tracker		
Fertilitäts App			menstrual period tracker		
Fruchtbarkeits App					
Familienplanungs App			cycle calculator		
			menstruation calculator		
Zyklus Applikation			menstrual calculator		
Menstruations Applikation			period calculator		
Perioden Applikation			ovulation calculator		
Ovulations Applikation			fertility calculator		
NFP Applikation			menstrual period calculator		
Fertilitäts Applikation					
Fruchtbarkeits Appliklation			digital menstruation calendar		
Familienplanungs Applikation			app based menstruation calendar		
			application based menstruation calendar		

Zyklustracker	digital menstruation diary
Menstruationstracker	app based menstruation diary
Periodentracker	application based menstruation diary
Ovulationstracker	digital menstrual calendar
NFPtracker	app based menstrual calendar
Fertilitätstracker	application based menstrual calendar
Fruchtbarkeitstracker	digital menstrual diary
Familienplanungstracker	app based menstrual diary
Eisprungtracker	application based menstrual diary
Zyklus Tracker	digital cycle diary
Menstruations Tracker	app based cycle diary
Perioden Tracker	application based cycle diary
Ovulations Tracker	digital cycle calendar
NFP Tracker	app based cycle calendar
Fertilitäts Tracker	application based cycle calendar
Fruchtbarkeits Tracker	
Familienplanungs Tracker	digital period diary
Eisprung Tracker	app based period diary
	application based period diary
digitaler Menstruationskalender	digital period calendar
digitaler Menstruations Kalender	app based period calendar
appbasierter Menstruationskalender	application based period calendar
appbasierter Menstruations Kalender	
applikationsbasierter Menstruationskalender	
applikationsbasierter Menstruations Kalender	
digitales Zyklustagebuch	
digitales Zyklus Tagebuch	
appbasiertes Zyklustagebuch	
appbasiertes Zyklus Tagebuch	
applikationsbasiertes Zyklustagebuch	
applikationsbasiertes Zyklus Tagebuch	
digitaler Eisprungkalender	
digitaler Eisprung Kalender	
appbasierter Eisprungkalender	
appbasierter Eisprung Kalender	
applikationsbasierter Eisprungkalender	
applikationsbasierter Eisprung Kalender	

Search Terms Combined

Zyklusapp	Kinderwunsch & Verhütung	Cycle app	Pregnancy planing & Contraception
Zyklusapp[tw] OR Menstruationsapp[tw] OR Periodenapp[tw] OR Ovulationsapp[tw] OR NFPApp[tw] OR Fertilitätsapp[tw] OR Fruchtbarkeitsapp[tw] OR Familienplanungsapp[tw] OR Zyklusapplikation[tw] OR Menstruationsapplikation[tw] OR Periodenapplikation[tw] OR NFPApplikation[tw] OR Fertilitätsapplikation[tw] OR Fruchtbarkeitsapplikation[tw] OR Familienplanungsapplikation[tw] OR Zyklus App[tw] OR Menstruations App[tw] OR Perioden App[tw] OR Ovulations App[tw] OR NFP App[tw] OR Fertilitäts App[tw] OR Fruchtbarkeits App[tw] OR Familienplanungs App[tw] OR Zyklus Applikation[tw] OR Menstruations Applikation[tw] OR Perioden Applikation[tw] OR Ovulations Applikation[tw] OR NFP Applikation[tw] OR Fertilitäts Applikation[tw] OR Fruchtbarkeits Appliklation[tw] OR Familienplanungs Applikation[tw] OR Zyklustracker[tw] OR Menstruationstracker[tw] OR Periodentracker[tw] OR Ovulationstracker[tw] OR NFPtracker[tw] OR Fertilitätstracker[tw] OR Fruchtbarkeitstracker[tw] OR Familienplanungstracker[tw] OR Eisprungtracker[tw] OR Zyklus Tracker[tw] OR Menstruations Tracker[tw] OR Perioden Tracker[tw] OR Ovulations Tracker[tw] OR NFP Tracker[tw] OR Fertilitäts Tracker[tw] OR Fruchtbarkeits Tracker[tw] OR Familienplanungs Tracker[tw] OR Eisprung Tracker[tw] OR digitaler Menstruationskalender[tw] OR digitaler Menstruations Kalender[tw] OR appbasierter Menstruationskalender[tw] OR appbasierter Menstruations Kalender[tw] OR applikationsbasierter Menstruationskalender[tw] OR applikationsbasierter Menstruations Kalender[tw] OR digitales Zyklustagebuch[tw] OR digitales Zyklus Tagebuch[tw] OR appbasiertes Zyklustagebuch[tw] OR appbasiertes Zyklus Tagebuch[tw] OR applikationsbasiertes Zyklustagebuch[tw] OR applikationsbasiertes Zyklus Tagebuch[tw] OR digitaler Eisprungkalender[tw] OR digitaler Eisprung Kalender[tw] OR appbasierter Eisprungkalender[tw] OR appbasierter Eisprung Kalender[tw] OR applikationsbasierter Eisprungkalender[tw] OR applikationsbasierter Eisprung Kalender[tw]	Fruchtbarkeit[tw] OR Fertiliät[tw] OR fertiles Fenster[tw] OR fruchtbare Tage[tw] OR Kinderwunsch[tw] OR Befruchtungsplanung[tw] OR Empfängnisplanung[tw] OR Schwangerschaftsplanung[tw] OR Familienplanung[tw] OR NFP[tw] OR Befruchtungs Planung[tw] OR Schwangerschafts Planung[tw] OR Familien Planung[tw] OR Schwangerschaft[tw] OR Verhütung[tw] OR Empfängnisverhütung[tw] OR Schwangerschaftsverhütung[tw] OR Empfängnisschutz[tw] OR Verhütungsmittel[tw] OR Verhütungsmethode[tw] OR Verhütungsplanung[tw] OR Verhütungsabsicht[tw] OR Vermeidung Schwangerschaft[tw] OR Empfängnis Verhütung[tw] OR Schwangerschafts Verhütung[tw] OR Verhütungs Mittel[tw] OR Verhütungs Methode[tw] OR Verhütungs Planung[tw] OR Verhütungs Absicht[tw] OR Schwangerschaftsvermeidung[tw]	cycle app[tw] OR menstruation app[tw] OR menstrual app[tw] OR period app[tw] OR ovulation app[tw] OR fertility app[tw] OR menstrual period app[tw] OR cycle application[tw] OR menstruation application[tw] OR menstrual application[tw] OR period application[tw] OR ovulation application[tw] OR fertility application[tw] OR menstrual period application[tw] OR cycle tracker[tw] OR menstruation tracker[tw] OR menstrual tracker[tw] OR period tracker[tw] OR ovulation tracker[tw] OR fertility tracker[tw] OR menstrual period tracker[tw] OR cycle calculator[tw] OR menstruation calculator [tw] OR menstrual calculator [tw] OR period calculator[tw] OR ovulation calculator[tw] OR fertility calculator[tw] OR menstrual period calculator[tw] OR digital menstruation calendar[tw] OR app based menstruation calendar[tw] OR application based menstruation calendar[tw] OR digital menstruation diary[tw] OR app based menstruation diary[tw] OR application based menstruation diary[tw] OR digital menstrual calendar[tw] OR app based menstrual calendar[tw] OR application based menstrual calendar[tw] OR digital menstrual diary[tw] OR app based menstrual diary[tw] OR application based menstrual diary[tw] OR digital cycle diary[tw] OR app based cycle diary[tw] OR application based cycle diary[tw] OR digital cycle calendar[tw] OR app based cycle calendar[tw] OR application based cycle calendar[tw] OR digital period diary[tw] OR app based period diary[tw] OR application based period diary[tw] OR digital period calendar[tw] OR app based period calendar[tw] OR application based period calendar[tw]	fertility[tw] OR fertile window[tw] OR fertile days[tw] OR desire to have a child[tw] OR fertilisation planning[tw] OR fertilization planning[tw] OR conception planning[tw] OR pregnancy planning[tw] OR family planning[tw] OR planned pregnancy[tw] OR ovulation prediction[tw] OR fertility awareness[tw] OR FABM[tw] OR planned parenthood[tw] OR reproductive planning[tw] OR intentional pregnancy[tw] OR pregnancy[tw] OR ovulation detection[tw] OR fertility monitoring[tw] OR rhythm method[tw] OR contraception[tw] OR birth control[tw] OR preventing pregnancy[tw] OR contraceptive[tw] OR avoid pregnancy[tw] OR pregnancy prevention[tw] OR fertility control[tw] OR birth prevention[tw] OR productive control[tw] OR prevent pregnancy[tw] OR contraceptives[tw] OR avoiding pregnancy[tw]

Entire Search String

((Zyklusapp[tw] OR Menstruationsapp[tw] OR Periodenapp[tw] OR Ovulationsapp[tw] OR NFPApp[tw] OR Fertilitätsapp[tw] OR Fruchtbarkeitsapp[tw] OR Familienplanungsapp[tw] OR Zyklusapplikation[tw] OR Menstruationsapplikation[tw] OR Periodenapplikation[tw] OR NFPApplikation[tw] OR Fertilitätsapplikation[tw] OR Fruchtbarkeitsapplikation[tw] OR Familienplanungsapplikation[tw] OR Zyklus App[tw] OR Menstruations App[tw] OR Perioden App[tw] OR Ovulations App[tw] OR NFP App[tw] OR Fertilitäts App[tw] OR Fruchtbarkeits App[tw] OR Familienplanungs App[tw] OR Zyklus Applikation[tw] OR Menstruations Applikation[tw] OR Perioden Applikation[tw] OR Ovulations Applikation[tw] OR NFP Applikation[tw] OR Fertilitäts Applikation[tw] OR Fruchtbarkeits Appliklation[tw] OR Familienplanungs Applikation[tw] OR Zyklustracker[tw] OR Menstruationstracker[tw] OR Periodentracker[tw] OR Ovulationstracker[tw] OR NFPtracker[tw] OR Fertilitätstracker[tw] OR Fruchtbarkeitstracker[tw] OR Familienplanungstracker[tw] OR Eisprungtracker[tw] OR Zyklus Tracker[tw] OR Menstruations Tracker[tw] OR Perioden Tracker[tw] OR Ovulations Tracker[tw] OR NFP Tracker[tw] OR Fertilitäts Tracker[tw] OR Fruchtbarkeits Tracker[tw] OR Familienplanungs Tracker[tw] OR Eisprung Tracker[tw] OR digitaler Menstruationskalender[tw] OR digitaler Menstruations Kalender[tw] OR appbasierter Menstruationskalender[tw] OR appbasierter Menstruations Kalender[tw] OR applikationsbasierter Menstruationskalender[tw] OR applikationsbasierter Menstruations Kalender[tw] OR digitales Zyklustagebuch[tw] OR digitales Zyklus Tagebuch[tw] OR appbasiertes Zyklustagebuch[tw] OR appbasiertes Zyklus Tagebuch[tw] OR applikationsbasiertes Zyklustagebuch[tw] OR applikationsbasiertes Zyklus Tagebuch[tw] OR digitaler Eisprungkalender[tw] OR digitaler Eisprung Kalender[tw] OR appbasierter Eisprungkalender[tw] OR appbasierter Eisprung Kalender[tw] OR applikationsbasierter Eisprungkalender[tw] OR applikationsbasierter Eisprung Kalender[tw]) AND (Fruchtbarkeit[tw] OR Fertiliät[tw] OR fertiles Fenster[tw] OR fruchtbare Tage[tw] OR Kinderwunsch[tw] OR Befruchtungsplanung[tw] OR Empfängnisplanung[tw] OR Schwangerschaftsplanung[tw] OR Familienplanung[tw] OR NFP[tw] OR Befruchtungs Planung[tw] OR Schwangerschafts Planung[tw] OR Familien Planung[tw] OR Schwangerschaft[tw] OR Verhütung[tw] OR Empfängnisverhütung[tw] OR Schwangerschaftsverhütung[tw] OR Empfängnisschutz[tw] OR Verhütungsmittel[tw] OR Verhütungsmethode[tw] OR Verhütungsplanung[tw] OR Verhütungsabsicht[tw] OR Vermeidung Schwangerschaft[tw] OR Empfängnis Verhütung[tw] OR Schwangerschafts Verhütung[tw] OR Verhütungs Mittel[tw] OR Verhütungs Methode[tw] OR Verhütungs Planung[tw] OR Verhütungs Absicht[tw] OR Schwangerschaftsvermeidung[tw])) OR ((cycle app[tw] OR menstruation app[tw] OR menstrual app[tw] OR period app[tw] OR ovulation app[tw] OR fertility app[tw] OR menstrual period app[tw] OR cycle application[tw] OR menstruation application[tw] OR menstrual application[tw] OR period application[tw] OR ovulation application[tw] OR fertility application[tw] OR menstrual period application[tw] OR cycle tracker[tw] OR menstruation tracker[tw] OR menstrual tracker[tw] OR period tracker[tw] OR ovulation tracker[tw] OR fertility tracker[tw] OR menstrual period tracker[tw] OR cycle calculator[tw] OR menstruation calculator [tw] OR menstrual calculator [tw] OR period calculator[tw] OR ovulation calculator[tw] OR fertility calculator[tw] OR menstrual period calculator[tw] OR digital menstruation calendar[tw] OR app based menstruation calendar[tw] OR application based menstruation calendar[tw] OR digital menstruation diary[tw] OR app based menstruation diary[tw] OR application based menstruation diary[tw] OR digital menstrual calendar[tw] OR app based menstrual calendar[tw] OR application based menstrual calendar[tw] OR digital menstrual diary[tw] OR app based menstrual diary[tw] OR application based menstrual diary[tw] OR digital cycle diary[tw] OR app based cycle diary[tw] OR application based cycle diary[tw] OR digital cycle calendar[tw] OR app based cycle calendar[tw] OR application based cycle calendar[tw] OR digital period diary[tw] OR app based period diary[tw] OR application based period diary[tw] OR digital period calendar[tw] OR app based period calendar[tw] OR application based period calendar[tw]) AND (fertility[tw] OR fertile window[tw] OR fertile days[tw] OR desire to have a child[tw] OR fertilisation planning[tw] OR fertilization planning[tw] OR conception planning[tw] OR pregnancy planning[tw] OR family planning[tw] OR planned pregnancy[tw] OR ovulation prediction[tw] OR fertility awareness[tw] OR FABM[tw] OR planned parenthood[tw] OR reproductive planning[tw] OR intentional pregnancy[tw] OR pregnancy[tw] OR ovulation detection[tw] OR fertility monitoring[tw] OR rhythm method[tw] OR contraception[tw] OR birth control[tw] OR preventing pregnancy[tw] OR contraceptive[tw] OR avoid pregnancy[tw] OR pregnancy prevention[tw] OR fertility control[tw] OR birth prevention[tw] OR productive control[tw] OR prevent pregnancy[tw] OR contraceptives[tw] OR avoiding pregnancy[tw]))
