# Supplementary material for: Reliability of Cycle Applications for Pregnancy Planning and Contraception: A Systematic Review
Source: Mayo Clin Proc Digit Health. 2025 Jun 9;3(4):100239. doi: 10.1016/j.mcpdig.2025.100239 (PMC12492232; doi:10.1016/j.mcpdig.2025.100239)
Supplement: Supplemental Appendix 4 [file mmc4.pdf]

Appendix 4: List of Abbreviations for Tables in Figures 2, 3 and 4

BL	Baseline
BT	Basal temperature
C	Contraception
ECO	Influence of confounding on the outcome reported
EL	Endline
FR	Comparison of the probability of becoming pregnant
HM	Hormone measurement (LH, estrone-3-glucuronide (E3G), progesterone, oestradiol) in blood or urine
IN	Interview
Mens	Menstruation
ML	Midline
N	Number of Participants
NoECO	No influence of confounding on the outcome reported
Ø	Average
OV	Ovulation
P	Participant
PI	Pearl Index
Prg	Pregnancy
Q	Questionnaire
SI	Sexual intercourse
Temp	Temperature
TTP	Time to pregnancy, Time to conception
US	Ultrasound examination
